# How the use of vaccines outside the cold chain or in controlled temperature chain contributes to improving immunization coverage in low- and middle-income countries (LMICs): A scoping review of the literature

**DOI:** 10.7189/jogh.11.04004

**Published:** 2021-01-31

**Authors:** Ibrahim K Dadari, Janice C Zgibor

**Affiliations:** 1 College of Public Health, University of South Florida, Tampa, Florida, USA; 2 United Nations Children’s Fund, Pacific Office, Solomon Islands

## Abstract

**Background:**

Most vaccines are recommended for storage at temperatures of +2°C to +8°C to maintain potency. Immunization supply chain bottlenecks constraints reaching populations with life-saving vaccines. The World Health Organization permits the use of vaccines outside the cold chain as “controlled temperature chain (CTC)” upon meeting certain conditions and has set targets to license more vaccines CTC by 2020.

**Objectives:**

This scoping review aims to explore and synthesize the evidence in the literature on how the use of vaccines outside the cold chain or in a controlled temperature chain increases immunization coverage in low and middle-income countries (LMICs), with a focus on the timelines of the Global Vaccine Action Plan (2011-2020).

**Methods:**

A systematic search of three online databases (PubMed, Embase, and Web of Science) due to their broad coverage of global health sciences retrieved 173 original peer-reviewed articles, of which 13 were included in the review having met our inclusion criteria.

**Results:**

The majority of the studies were conducted in Africa (n = 9), followed by Asia (n = 3), and the least in the Pacific (n = 1). The different study designs captured included four non-randomized trials, three randomized trials, two simulation models, two cross-sectional studies, and one cohort study. Reported benefits included increased coverage, logistical ease, cost savings while vaccines remain potent.

**Conclusion:**

Currently, only two vaccines have been licensed to be stored CTC. More needs to be done to get additional vaccines licensed for CTC and disseminate operational guidance to operationalize its use in low- and middle-income countries.

Recently, there is an increase in the use and, or storage of vaccines at room temperature or above the recommended storage values to increase immunization coverage. Most vaccines are stored at temperatures of +2 to +8°C referred to as the 'cold chain', to maintain potency [1,2]. Despite these recommendations, there are identified weaknesses in maintaining the vaccine ‘cold chain’ in both developed and developing countries with demonstrable evidence of exposure to freezing temperatures, high temperatures above recommended, and other avoidable errors that are not experienced in more developed countries [3]. While it may be erroneous to expose most vaccines to temperatures outside the recommended +2 to +8°C, challenges in maintaining the cold chain, especially in low- and middle-income countries, coupled with recent advances in vaccine development and delivery, may allow for the use of some vaccines stored at higher or room temperatures under proper monitoring.

The World Health Organization (WHO) [4] recommends the use of vaccines under a ‘controlled temperature chain’ (CTC), which is an innovative approach of keeping vaccines outside the recommended +2 to +8°C, under proper monitoring for a limited time before administration. The WHO (2018) set two conditions for the CTC which include: the vaccines can withstand room temperatures up to +40°C, and these vaccines should be used for campaigns or special delivery and not routine immunization. The MenAfriVac (Meningitis A) vaccine was the first to be licensed for CTC delivery in 2012 [5]. Other vaccines, which are yet to be labeled CTC, such as Hepatitis B (HepB), remain potent and effective when stored at room temperatures [6]. The use of these vaccines outside the recommended temperatures is considered an “off-label” use for which the persons allowing the user must take responsibility that it is appropriately monitored [7].

To accelerate the successes being recorded for the immunization program and achieve the ambitious vision of the Decade of Vaccines Collaboration, which stipulates universal access to immunization [8], a Global Vaccines Action Plan (GVAP) 2011 to 2020 was endorsed by 194 member states at the May 2012 World Health Assembly [8]. Among the many strategies the GVAP is adopting to achieve universal access to immunization, use of vaccines in CTC or above the recommended temperatures of +2 to +8°C is being measured under indicator 6.4 of the GVAP – Number of vaccines that have either been re-licensed or licensed for use in a controlled-temperature chain at temperatures above the traditional 2-8°C range [9]. The CTC – Working Group was constituted and endorsed by the WHO Immunization Practices Advisory Group in February 2016, to accelerate achieving higher vaccine coverage and equity targets through a strategic roadmap [4]. Four vaccines were considered a priority by the CTC roadmap including Human papillomavirus (HPV), Oral cholera vaccine (OCV), TT-CVs [tetanus-toxoid-containing vaccines], and Hepatitis B vaccine birth dose (HepB-BD) [9].

Findings from a multi-level stakeholder interview in six low- and middle-income countries regarding the benefit of using thermostable vaccines showed the majority were willing to pay a bit more to get thermostable vaccines which will assist them in addressing the persistent and recurring challenges of the immunization supply chain systems in their respective countries [10]. According to Kristensen et al., over 70% of interviewed stakeholders from six countries in the study expressed their interests in CTC labeled vaccines, acknowledging the flexibility this brings to the program but also expressing concerns regarding the possible confusion which may arise from changing the storage conditions for some and not all the vaccines [10].

The key objectives of this scoping review paper include to synthesize and explore evidence from the literature on the use of vaccines outside the cold chain or in a controlled temperature chain across low and middle-income countries (LMICs), with particular focus on the period spanning the decade of vaccines collaboration and timelines of the GVAP, and how this use contributes to increasing immunization coverage and equity across these countries. Furthermore, this scoping review will help identify any relevant policy and implementation gaps towards achieving the strategic objectives of the GVAP in getting vaccines licensed and implemented as CTC. As such, the following research question was formulated: How the use of vaccines outside the cold chain or in a controlled temperature chain contributes to improving immunization coverage in low- and middle-income countries (LMICs)?

To the best of our knowledge, this will be the first scoping review looking at CTC or vaccines use outside the cold chain globally, more particularly as it relates to the decade of vaccines 2011 to 2020, which also aligns with the timelines of the Global Vaccine Action Plan (GVAP). As a result, the combination of search terms and search strategy used are originally designed for this review. Findings will inform evidence about lessons learned and best practices especially with regards to increasing vaccination coverage using these strategies.

## METHODOLOGY

This study is conducted following the updated methodology for the conduct of scoping reviews published by the Joanna Briggs Institute (JBI) [11] building on the seminal work of Arksey and O’Malley [12], and reporting guided by the Preferred Reporting Items for Systematic Reviews and Meta-analysis extension for Scoping Reviews (PRISMA-ScR) checklist (Appendix S1 in the **Online Supplementary Document**) [13], and the PRISMA-S extension for reporting searches in systematic reviews (Appendix S2 in the **Online Supplementary Document**) [14]. To be included in this review, relevant original peer-reviewed journal articles must meet the context and content criteria of this scoping review and published between the years 2011 and 2020 which is the duration of the GVAP and the Decade of Vaccines. As such, the gray literature not meeting the inclusion criteria for this review was not included in this search.

### Search strategy

This literature search was conducted in a multistep approach where three databases were identified for the search due to their broad coverage of global health sciences. The search strategy was developed by the first author (I.K.D) of this paper with first review and inputs provided by an experienced librarian (A.H) at the University of South Florida (USF) College of Public Health, while final review and approval of the search strategy was provided by the second author (J.C.Z). The screening of search results, review of title and abstracts was an iterative process between the two authors of this paper with inputs from other faculty. The final search strategy is presented in Appendix S3 in the **Online Supplementary Document**. All article and publication types were searched in the following three databases including PubMed, Embase, and Web of Science using the following major concepts: (“outside the cold chain” OR “controlled temperature chain” OR “thermostable”) AND (“immunization coverage” OR “vaccination coverage”) AND (“low middle-income countries” OR “developing countries”). Synonyms and controlled vocabularies were used for the search. Article search was limited to publication years 2011 to 2020 to align with the timelines of the Global Vaccine Action Plan (GVAP) to help in assessing evidence available in support of indicator 6.4. – number of vaccines that have either been re-licensed or licensed for use in a controlled-temperature chain at temperatures above the traditional +2 to +8°C range [9]. Literature search was limited to above three databases for their broad coverage of global health sciences with no simultaneous or single platform database searches conducted. Citing references were examined in the process with zero yield. In the process of defining search terms and search strategies, some purposeful searching was conducted to finetune the search strategy, however no purposeful search items were included in the final search. No search was conducted on study registries or by contacting other external sources.

### Inclusion and exclusion criteria

Articles retrieved were critically evaluated to be included in the review if they met the following criteria:

- Original research articles focused on vaccines and immunization in low- and middle-income countries
- Focus on vaccine use outside the recommended temperatures of +2 to +8°C. This included thermostable vaccines, vaccines used off-label outside the cold chain, and controlled temperature chain (CTC) vaccines.
- Articles written between the years 2011 and 2020 (decade of vaccines) coinciding with the timelines of the Global Vaccines Action Plan.
- Studies with relevant information on coverage indices applied to real-life situations.

Articles were excluded if they were reviews, commentaries, opinion or perspective articles, animal studies, early clinical trials (Phase I & II), or studies not conducted in low- and middle-income countries.

### Articles retrieved

**Figure 1:**
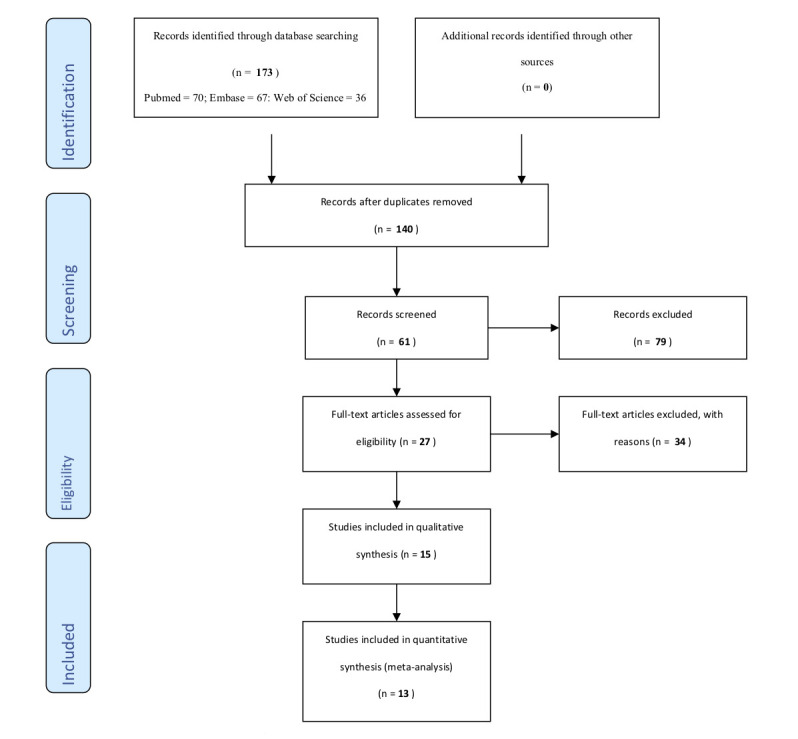
PRISMA 2009 flow diagram.

A total of 173 articles were retrieved from the search (PubMed = 70, Web of Science = 36, and Embase = 67) (**Figure 1**). Articles were exported to EndNote library and referencing software where duplicates were removed retaining a combined total of 140 articles. A Title/Abstract screen was conducted on the articles identifying articles relevant to immunization, which resulted in retaining 61 articles. Following a full-text review, 27 articles were retained as relevant to the use of vaccines outside the cold chain or CTC. Twelve of the 27 articles were excluded because they did not meet the inclusion criteria listed above. The remaining 15 were further screened leading to the removal of two articles noted to be very similar to two other studies, but published in different journals, so they were excluded. A final 13 articles were included in the review. This search was conducted on the 2nd of March 2020.

### Data extraction and management

Data was extracted using a template designed for this scoping (**Table 1**, **Table 2**), and the process was iterative between the two authors. Specific article elements and information based on their relevance to the scope and objectives of this review were included in the table. Article characteristics extracted included (a) authors (b) publication year (c) topic title and focus (d) study setting (e) population selection (f) study design and methodology (g) relevant key findings, and (h) study limitations. A basic descriptive analysis of the data was conducted with a focus on the following themes (a) controlled temperature chain (CTC) vaccination (b) use of vaccines outside the cold chain (OCC) (c) effect on immunization coverage (d) vaccine potency and immunogenicity (e) vaccine wastage (f) temperature excursions (g) adverse events following immunization (AEFI) (h) cost savings and economic benefits, and (i) other benefits. We did not perform a critical appraisal or risk of bias assessment, as this is generally not recommended for scoping reviews [11,13,28].

**Table 1:** Summary of study characteristics included in the systematic review to assess how the use of vaccines outside the cold chain or in controlled temperature chain contributes to improving immunization coverage in low- and middle-income countries (LMICs)

Author(s), year	Topic/Focus	Study setting	Population selection	Study design/Methodology
Zipursky et al., 2011 [15]	“Assessing the potency of oral polio vaccine kept outside of the cold chain during a national immunization campaign in Chad.” This study systematically investigated the potency of the mOPV3 used during the campaign in Chad and exposed to ambient temperatures that were still potent.	N’djamena, Chad (LMIC)	22 OPV vials selected at random -20 test vials & 2 control vials of OPV used	**Randomized Controlled Trial** 20 test vials labeled T1-T20 and 2 control vials labeled C1 & C2 were exposed to room temperature. A data logger was used to monitor temperature exposure and a visual percentage-based color intensity classification scale with values ranging from 0% to 100% (perfect VVM to 100% unusable). Laboratory analysis to assess potency was conducted on the vials.
Lee et al., 2012 [16]	“The impact of making vaccines thermostable in Niger's vaccine supply chain”.	Niger (LMIC)	1 central store, 8 regional stores, 42 district stores, and 695 clinics.	**Computational, discrete-event simulation model** using the HERMES framework.
Shrivastava, et al., 2012 [17]	“Caution needed in using oral polio vaccine beyond the cold chain: vaccine vial monitors may be unreliable at high temperatures”.	India (LMIC)	Uttar Pradesh and Bihar States in India. (National Institute of Immunology, New Delhi)	**Cohort Study** 10 vials each of OPV vaccines with VVM lids were incubated at 37, 41, 45, and 49.5°C in a dry incubator. VVMs monitored hourly and examined by independent observers.
Juan-Giner et al., 2014 [18]	“A cluster randomized non-inferiority field trial on the immunogenicity and safety of tetanus toxoid vaccine kept in controlled temperature chain compared to cold chain”. The aim was to demonstrate the potency of TT kept in CTC compared to SCC.	Moïssala district in Chad (LMIC)	2128 participants from 42 villages grouped into 34 clusters were enrolled (1068 in CTC; 1060 in SCC) with 952 completing the study in each group.	**Cluster-Randomized Non-Inferiority Field Trial** The thirty-four clusters were randomized to CTC or SCC. TT in 10 dose-vials were kept at room temperature inside a vaccine carrier for 30 days before mass administration. Women aged 14-49 years, eligible for TT vaccination, received 2 doses of TT and antibody titers taken.
Lydon et al., 2014 [19]	“Economic benefits of keeping vaccines at ambient temperature during mass vaccination: the case of meningitis A vaccine in Chad”.	N'Djamena; Chari Baguirmi and Mayo Kebbi East regions of Chad (LMIC)	1 807 158 individuals vaccinated in December 2011 across 12 districts in the three regions.	**Cross-sectional study + mathematical modeling of cost** Qualitative information (through interviews and on-site observations) and quantitative data drawn from a variety of primary and secondary sources.
Steffen, et al., 2014 [20]	“A field-based evaluation of adverse events following menafrivac® vaccine delivered in a controlled temperature chain (CTC) approach in Benin.”	Eight villages in Benin Republic (LMIC)	1000 participants were included in the CTC and 999 in the non-CTC group from 630 households.	**Non-randomized Controlled trial** Following vaccination with MenAfriVac household survey data was collected in five consecutive days starting a day or two days after vaccination where the campaign lasted a single day or several days respectively.
Zipursky, et al., 2014 [21]	“Benefits of using vaccines out of the cold chain: delivering meningitis A vaccine in a controlled temperature chain during the mass immunization campaign in Benin”	One district in Benin Republic (LMIC)	21 supervisors and 77 vaccinators were surveyed in one district, with a target population of 147 207; 1-29 years of age.	**Cross-sectional study** The fixed site and mobile/outreach teams to vaccinate the population using CTC. Heat sensitive sticker used for temperature monitoring (changes when 40°C+).
Kolwaite et al., 2016 [22]	“Hepatitis B vaccine stored outside the cold chain setting: a pilot study in rural Lao PDR”.	Four districts Lao PDR (LMIC)	388 children aged 2-8 months and 371 children aged 14-20 months were enrolled in intervention districts; 190 children aged 2-8 months and 184 children aged 14-20 months were enrolled in the comparison districts.	**Non-randomized Controlled trial** Healthcare workers in intervention districts were trained to store two-dose monovalent HepB* vaccine vials in ambient temperatures for up to 28 days. After 28 days, the unused HepB vaccine was discarded. HepB vaccine was stored in a standard cold chain in comparison. Standardized questionnaire to monitor adverse events following immunization (AEFI).
Breakwell, et al., 2017 [23]	“Evaluation of storing hepatitis B vaccine outside the cold chain in the Solomon Islands: Identifying opportunities and barriers to implementation”.	Three provinces in the Solomon Islands (LMIC)	364 births, of which 278 (76%) were HF births.	**Non-randomized trial** Healthcare workers at 13 facilities maintained monovalent HepB birth dose (HepB-BD) OCC for up to 28 days over 7 months. Temperature excursions monitored with Log Tags.
Landoh et al., 2017 [24]	“Impact of Controlled Temperature Chain (CTC) approach on immunization coverage achieved during the preventive vaccination campaign against meningitis A using MenAfriVac in Togo in 2014”.	4 regions in Togo (LMIC)	A total of 2707 households were surveyed, and 9082 people aged 1-29 years were interviewed in 4 of 10 regions that implemented CTC.	**Cross-sectional survey** with interviews. Two-stage cluster sampling was used with multivariate analysis was used to analyze data.
Lee et al., 2017 [25]	“Economic impact of thermostable vaccines” using a computational model with data from three countries.	Republic of Benin, the state of Bihar (India), and Niger republic. (LMICs)	Data from national and many subnational stores in the three places.	**HERMES simulation models** replacing different vaccines in the supply chain of the Republic of Benin, the state of Bihar (India), and Niger. The resulting clinical and economic impacts were estimated.
Mvundura et al., 2017 [26]	“An economic evaluation of the controlled temperature chain approach for vaccine logistics: evidence from a study conducted during a meningitis A vaccine campaign in Togo”.	Four districts in Togo (LMIC)	22 sites were included in the data collection: national level (n = 1), regional level (n = 1), districts (n = 4), and health centers (n = 16).	**Non-randomized trial** 2 districts used the CTC approach, and 2 districts used the standard storage (full CCL) approach during the MenAfriVac campaign in 2014.
Coldiron et al., 2018 [27]	“Safety of a heat-stable rotavirus vaccine among children in Niger: Data from a phase 3, randomized, double-blind, placebo-controlled trial”. The primary outcome of the trial was efficacy against SRVGE.”	Madarounfa District in Niger Republic (LMIC)	3680 in the RotaSIIL group and 3705 in the placebo group.	**Randomized Control Trial** Children were randomized to receive RotaSIIL or placebo at 6, 10, and 14 weeks of age and were followed up for two years. Stool samples were taken within seven days of a reported gastroenteritis episode and tested for rotavirus antigen. χ^2^ and Fisher exact tests done.

LMIC – low- and middle-income country, SRVGE – severe rotavirus gastroenteritis, CTC – controlled temperature chain, AEFI – adverse events following immunization, HepB-BD – hepatitis B birth dose, VVM – vaccine vial monitor, SCC – standard cold chain storage, TT – tetanus toxoid, HERMES - highly extensible resource for modeling supply chains, HF – health facility, HepB – hepatitis B, OPV – oral polio vaccine, CCL – cold chain logistics, OCC – outside the cold chain

*Hepatitis B Vaccine (HepB) from Shanvac®-B, Shantha Biotechnics Private Ltd, India.

**Table 2:** Summary of key findings and limitations of the studies included in the systematic review to assess how the use of vaccines outside the cold chain or in controlled temperature chain contributes to improving immunization coverage in low- and middle-income countries (LMICs)

Author(s) Year	Relevant key findings	Study limitations.
Zipursky et al., 2011 [15]	The use of a CTC-based strategy allowed for vaccination to happen in areas with unreliable cold chain proffers logistical and operational advantages including allowing health workers to conduct home vaccination anytime. Health Workers were comfortable, but the parents were not. Ambient temperature exposure for the vials reached a maximum of 47.1°C. A total of 6 vials reached the VVM discard point at end of day 2.	Vaccines used in the study were from the same batch and manufacturer, and the study did not allow for a detailed correlation to be drawn between the length of time and temperature exposure and vaccine potency reached and VVMs.
Lee et al., 2012 [16]	Making vaccines thermostable had positive effects by reducing supply chain bottlenecks and increase the availability of all EPI vaccines and decreased cold chain space utilization. Thermostable pentavalent had the highest positive effect with its availability increasing from 87% to 97%, and the availability of other non-thermostable EPI vaccines increased to over 93%.	A study is a Model that cannot capture every detail of reality. Assumptions that the physical characteristics of the vaccine remain the same when made thermostable.
Shrivastava, et al., 2012 [17]	The 10 test vials reached the discard point in 43 hours at 37°C storage, 24 hours at 41°C storage, 16 hours at 45°C, and 9 hours at 49.5°C storage temperatures. Findings suggest VVMs are not reliable indicators of vaccine potency at high environmental temperatures.	The study did not conduct a laboratory test for vaccine potency.
Juan-Giner et al., 2014 [18]	Following vaccination, overall seroprotection was the same in both groups; 99.34% in the CTC and 99.45% in the SCC groups. Few adverse events were noted. Thus, the study demonstrated immunogenicity and safety of TT vaccines in CTC at <40°C for <30 days. Maximum recorded ambient temperature of 43.1°C with no damage to vaccines from heat exposure.	Only vaccines from one manufacturer were used for the study – Serum Institute India.
Lydon et al., 2014 [19]	The study demonstrated large cost savings that could be obtained when vaccines are kept at CTC more especially from district-level storage down to service delivery points.	Used a single scenario in the model, could not estimate additional costs of CTC, and data not generalizable.
Steffen, et al., 2014 [20]	Incidence rates of AEFIs in the CTC group were the same or less than those in the non-CTC group (No hospitalizations record). Ambient temperatures ranged from 19°C to 46°C during the vaccination period.	Non-randomization and a non-representative population sample.
Zipursky, et al., 2014 [21]	An overall vaccination coverage of 105.7% was achieved (155 596 people vaccinated) with vaccines stored under three different CTC scenarios. All vaccinators and 98% of supervisors' preference to use CTC for the next campaign. No VVMs reached the discard point, and no temperature reading up to 40°C. Minimal challenge with CTC.	The sample was not representative of the population.
Kolwaite et al., 2016 [22]	A 27% median increase (interquartile range [IQR] 58%, *P* < 0.0001) in HepB-BD coverage in the intervention districts, compared with a 0% median change (IQR 25%, *P* = 0.03) in comparison districts. No adverse reactions were reported. Median temperature exposure for the vaccine was 27°C in the intervention group and 4.6°C in the control group.	Several health facilities were not enrolled due to access issues and some selected villages only had children from one age group, which made a comparison impossible.
Breakwell, et al., 2017 [23]	Timely HepB-BD vaccination coverage increased from 30% (n = 38/125) to 68% (n = 104/152) (*P* = 0.0005) and from 4% (n = 2/46) to 24% (n = 9/38) among facility and home births respectively. Additionally, BCG 24-h coverage increased from 15% (n = 19/125) to 28%. By 42 d post-birth, HepB-BD coverage had reached 80% (n = 121/152). Rarely temperatures exceeded 37°C, but vaccine wastage was high and shortages common. Where home births are common, an outside cold chain policy could improve birth dose coverage.	Some health facilities had very low births and sample not representative of the total population.
Landoh et al., 2017 [24]	No statistical differences in vaccination coverage between CTC and non-CTC areas (AOR = 0.09; 95%CI = -0.27, 0.45). The overall vaccination rate was 98% of the surveyed population. Mild to moderate AEFI in 2.3% following vaccine administration.	A non-representative sample of the regions was taken.
Lee et al., 2017 [25]	Replacing a particular vaccine with a thermostable version yielded cost savings in many cases even when charging a price premium. For instance, replacing the current pentavalent vaccine with a thermostable version with or without increasing the vaccine price was cost-saving (US$366 to US$10 945 per 100 members of the vaccine's target population). Cost savings observed even when vaccine prices were doubled or tripled.	The study is a model and may not capture all real-life scenarios. Model assumed vaccine will maintain the same character when it becomes thermostable.
Mvundura et al., 2017 [26]	The cost of logistics per dose administered was not statistically different between CTC and standard storage. There is a possibility of increased cost per dose if the facilities without refrigerators had not used a CTC. The analysis showed that the strongest case for CTC use is for remote health centers without cold chain equipment.	Possibly underestimated the cold chain costs because the costs at the regional level were not included in the analysis. Transport costs may have been under-budgeted.
Coldiron et al., 2018 [27]	Children who received the RotaSIIL vaccine had similar safety outcomes compared to placebo. Only one case of intussusception reported.107 deaths split near half among the two groups. SAEs occurred in 814 participants, 395 (19.3%) RotaSIIL and 419 (20.5%) control. A total of 7385 child-years of follow-up.	The complexity of making an accurate diagnosis of intussusception in remote settings.

SAE – serious adverse effects, CTC – controlled temperature chain, AEFI – adverse events following immunization, HepB-BD – hepatitis B birth dose, BCG – bacillus Calmette Guerin, CI – confidence interval, AOR – adjusted odds ratio, EPI – expanded programme on immunization, VVM – vaccine vial monitor, SCC – standard cold chain storage, TT – tetanus toxoid, IQR – interquartile range

## RESULTS

Evidence from the literature shows the actual and potential benefits of thermostable vaccines, storing vaccines outside the cold chain (OCC), or the use of vaccines approved for controlled temperature chain (CTC) in low- and middle-income countries. Of the total 173 articles retrieved in the search, 13 were included in this review having met all the inclusion criteria of the study. For each of the studies, the authors, study title, study setting, population selection, study design, relevant key findings, and study limitations were charted. The different study designs captured included four non-randomized trials [20,22,23,26], three randomized trials [15,18,27], two simulation models [16,25], two cross-sectional studies [19,21], and one cohort study [17]. The majority of the studies were conducted in Africa (n = 9), followed by Asia (n = 3), and the least in the Pacific (n = 1). Three of the studies used country data to model potential coverage, supply chain, and economic impacts of storing and using vaccines outside the standard cold chain storage [16,19,25]. Two studies looked at the increased immunization coverage and other benefits of ‘off-label’ use of vaccines (Hepatitis B) outside the cold chain [22,23]. So far MenAfriVac vaccine, the first to be licensed by the WHO for use in ambient temperatures subject to meeting certain conditions – controlled temperature chain; has been extensively studied [19,20,24]. The study characteristics are summarized in **Table 1**, and the key findings and limitations are summarized in **Table 2**.

### Controlled temperature chain (CTC) vaccination

Six of the articles in this review studied and reported on the (potential) benefits or otherwise of keeping vaccines under CTC conditions [18-21,24,26]. Two of the studies were non-randomized trials with one that explored the economic evaluation of the controlled temperature chain approach for vaccine logistics during the meningitis A vaccine campaign in Togo using CTC vaccines, while the other was a field-based evaluation of adverse events following immunization (AEFI) from the same MenAfriVac (CTC) mass campaign conducted in Benin republic [20,26]; two articles were cross-sectional studies with one being an immunization coverage survey that assessed the impact of the vaccine administered CTC in Togo in 2014, and the second study examined the benefits of delivering Meningitis A (MenAfriVac) vaccines in a controlled temperature chain in the Benin Republic following a mass immunization campaign [21,24]; and one study used cross-sectional study data to model the economic benefits of MenAfriVac vaccine stored CTC in three regions of Chad republic where over 1.8 million eligible were vaccinated in December 2011 [19].

### Use of vaccines outside the cold chain (OCC)

The off-label use of Hepatitis B vaccines outside the cold chain was studied and reported by Kolwaite et al. [22] and Breakwell et al. [23]. Both studies were non-randomized trials conducted in select populations at the subnational level in Lao PDR and the Solomon Islands respectively, looking at the benefits of storing and using Hepatitis B vaccine outside the cold chain in these countries. In both studies, the monovalent Hepatitis B vaccine was stored at room temperature for 28 days before being discarded. Within those 28 days, for which the HepB vaccine was considered potent, children were vaccinated as per the national expanded program on immunization (EPI) schedule of each of the countries. While the study in Lao PDR enrolled two cohorts of children; 388 children aged 2-8 months and 317 children aged 14-20 months; the Solomon Island study enrolled 364 children from birth to improve the coverage of HepB birth dose among these children. Both studies used a control to compare the benefits of OCC. Zipursky and colleagues [15] reported a study that systematically assessed the potency of monovalent oral polio vaccine (mOPV3) kept outside of the cold chain and exposed to room temperatures during a national immunization campaign in N’djamena the capital of Chad republic. Another study conducted in Niger republic was a modeling study using discrete-event simulation models with the Highly Extensible Resource for Modeling Supply Chains (HERMES) framework to assess the impact of making vaccines thermostable in the immunization supply chain, where the authors conducted the simulation using data collected from one central store, 8 regional vaccine stores, 42 district stores and 695 health centers in the country [16]. Findings from this study showed a net positive effect on the supply chain if vaccines are made thermostable, thereby increasing vaccine availability, reduced cold chain requirements, and reducing supply chain bottlenecks.

Another study assessed the immunogenicity and safety of tetanus toxoid (TT) vaccine kept in ambient temperatures in the Chad republic which is considered as an OCC storage [18]. This study was a cluster-randomized non-inferiority trial that compared TT vaccines kept in the standard cold chain (SCC) conditions as against the same vaccines kept outside the cold chain in Moissala, Chad republic.

### Effect on immunization coverage

The majority of these studies assessed the effect of using vaccines stored OCC or CTC and showed increased vaccination coverage with the use of these strategies. Out of the 9000 eligible persons aged 1-29 years who were surveyed by Landoh et al. [24], a very high immunization coverage was observed with about 98% of persons reporting being vaccinated. While the survey was conducted on a non-representative sample of the population, no statistically significant difference in vaccination coverage between CTC and non-CTC areas was observed (adjusted Odds Ratio AOR = 0.09; 95% confidence interval CI = [-0.27-0.45]). A non-representative sample survey of individuals eligible for vaccination following the mass campaign in Benin using CTC showed a very high immunization coverage of 105.7% achieved amongst the targeted population as reported by Zipursky et al. [21]. An immunization coverage of >100% as seen here could be due to factors such as vaccination of age groups outside the target population or data quality issues. Kolwaite et al. [22] were able to show from their HepB OCC pilot in Lao PDR, a 27% median increase in Hepatitis B birth dose (HepB-BD) coverage (interquartile range [IQR] 58%, *P* < 0.0001) observed in the pilot clinics as compared to pre-pilot HepB-BD coverage in the same district. Whereas there was no change (IQR 25%, *P* = 0.03) in comparison districts observed. From a similar pilot conducted in the Solomon Islands, Breakwell et al. observed an increase in timely HepB-BD coverage from 30% in a comparable pre-pilot period to 68% among health facility deliveries and an increase in coverage from 4% to 24% among children delivered at home in the intervention catchment [23]. By 42 days post-birth, HepB-BD coverage reached a high of 80% (n = 121/152) during the pilot period with 85% (n = 104/122) of the vaccinations being timely HepB-BD (within 24% of birth). Additionally, in the same study, BCG coverage within 24hours of birth increased from 15% (n = 19/125) to 28% which was an unintended benefit of the OCC. The modeling study by Lee et al. showed that making pentavalent vaccines thermostable increased its availability from 87% to 97%, and increased availability of other non-thermostable vaccines to 93% [25]. Further making Yellow Fever (YF) and Tetanus Toxoid (TT) vaccines thermostable resulted in a 1–2% increased availability of all EPI vaccines, while other vaccines when made thermostable showed marginal or no observed benefits. In this setting, making pentavalent vaccine thermostable showed the highest benefit of increasing vaccine availability as compared to other vaccines.

### Vaccine potency and immunogenicity

Juan-Giner and colleagues [18] reported a randomized study involving 2128 Women aged 14–49 years, residing in 42 villages grouped into 34 clusters and eligible for TT vaccination with a history of ≤1 TT dose were enrolled, blinded, and randomized into CTC and SCC groups (1068 in CTC and 1060 in SCC). Tetanus toxoid vaccines stored outside the cold chain and exposed to temperatures between 21.4 and 38.3°C, with a maximum reported temperature exposure being 43.1°C over four weeks showed a similar seroprotection among the two groups of women: 99.34% in the CTC and 99.45%. The researchers concluded based on these findings that TT vaccines stored at <40°C for <30 days were safe and immunogenic.

A study conducted in the states of Uttar Pradesh and Bihar in India to check the reliability of vaccine vial monitors (VVM) at high temperatures in Uttar Pradesh and Bihar states of India, suggested that VVM indicators were not reliable at high ambient temperatures with the incubated vials reaching discard point as follows; at 37°C all 10 vials reached discard point within 43hours, 24hours for 41°C, 16 hours for 45°C and 9 hours for all the ten vials at 49.5°C [17]. A key limitation of this study is that vaccine potency was not assessed.

### Vaccine wastage

One of the factors assessed by the authors in the different studies was vaccine wastage which means vaccines that are discarded and not administered to eligible populations for any reason. Most studies did not indicate high vaccine wastage except one, which could be attributed to the small sparse population and no baseline to compare whether the pre-intervention period had a similarly high vaccine wastage rate [23]. The pilot study on the use of HepB OCC in the Solomon Islands showed high vaccine wastage rates [23]. Despite this, it was concluded that outside the cold chain policy will be very useful in situations where home births are common, which is underpinned by regular vaccine supply and less vaccine wastage.

Zipursky and colleagues [15] assessed the potency of oral polio vaccine kept outside of the cold chain during a national immunization campaign in Chad and reported temperature exposures above 8°C for test vials that spent 1 day outside between 10.7 and 24.6 hours, and for vials that spent 2 days outside lasting between 17.8 and 86.9 hours. While only one vial reached the VVM discard point at the end of day 1, five test vials reached VVM discard point at the end of day 2. In another study, Zipursky et al. [21] discovered no vaccine reached VVM Stages 3 or 4 (discard point) after storing outside the cold chain, and no vial was discarded due to very high-temperature excursions (>40°C).

### Temperature excursions

While storing vaccines outside the cold chain or in a controlled temperature chain, occasional exposures to very high ambient temperatures do occur. Even as vaccines were exposed to ambient temperatures, swings above 40°C were rare and few vaccines were discarded due to VVM change from extremes of temperature [15,18,22,23]. The study from Lao PDR by Kolwaite et al. [22] showed a median temperature excursion for HepB vaccines stored OCC as compared to comparison clinics where the vaccine was stored at recommended cold chain storage of 4.6°C and 27°C respectively. The maximum temperature excursion in the field trial conducted to demonstrate the immunogenicity and safety of the tetanus toxoid (TT) vaccine stored outside the cold chain was 43.1°C, with a range of between 21.4 and 38.3°C [18]. The pilot study from the Solomon Islands showed a maximum recorded spike in temperature exposure for the vaccines was 46.6°C, with temperature excursions above 37°C rare [23]. The authors concluded that outside the cold chain policy will be very useful in situations where home births are common, which is underpinned by regular vaccine supply and fewer vaccine wastages.

### Adverse Events Following Immunization (AEFIs)

One of the potential consequences of improper vaccine storage could be an increase in the incidence of adverse events following immunization (AEFI) among recipients of the vaccine. Kolwaite et al. [22] reported no adverse events in their study in Laos PDR. From the field-based evaluation of adverse events following immunization (AEFI) conducted by Steffen et al. [20] for the MenAfriVac mass campaign in Benin republic, there was no statistically significant difference in the incidence rates of AEFIs among the CTC and the non-CTC population. Landoh et al. [24] in their cross-sectional study conducted in four of the ten regions that implemented CTC in Togo in 2014, reported a 2.3% incidence of mild to moderate AEFIs among study participants which included fever, abscesses, and swelling at the injection site. Coldiron and colleagues [27] having conducted a study randomizing of 4092 children to receive either RotaSIIL or placebo at 6, 10 and 14 weeks of age, with 2 years follow up, recorded only one case of intussusception 542 days after the third dose of RotaSIIL; a total of 107 deaths occurred split near half among the two groups, Serious Adverse Events (SAEs) occurred in 814 participants; 395 (19.3%) who had received RotaSIIL and 419 (20.5%) in the control group. Coldiron and colleagues [27] reported the more common causes of SAEs to include malaria, lower respiratory tract infections, gastroenteritis, and marasmus. While there were mostly no significant differences in SAEs between the control and intervention groups, more cases of lower respiratory tract infections were reported in the control group as compared to the intervention group who received RotaSIIL (8.8% vs 6.9%, *P* = 0.02). Overall findings from the study showed that children who received RotaSIIL had similar safety outcomes as compared to children who received a placebo. But this study has the limitation of the complexity of making an accurate diagnosis of intussusception especially in remote locations in the Niger republic.

### Cost savings and economic benefits

Lydon et al. [19] studied the economic benefits of vaccines kept CTC, with findings showing substantial cost savings if vaccines are kept and used CTC, particularly from the district level downwards. This cost savings assumed no loss of vaccine potency, efficacy, or safety. An economic evaluation of the controlled temperature chain approach for vaccine logistics during the meningitis A vaccine campaign in Togo, where two of the districts (Sotouboua and Britta) vaccines were stored CTC while in the other two districts (Tchaoudjo and Tchamba) vaccines were kept in standard vaccine storage at +2 to +8°C showed the cost of vaccine logistics per dose administered was not significantly different between standard vaccine storage at recommended temperature vs vaccines stored in a controlled temperature chain [26]. However, the analysis showed a possibility of an increased cost per vaccine dose should health facilities without cold chain equipment or refrigerators fail to use the CTC vaccine. From this, Mvundura and colleagues concluded that the strongest case for the use of CTC in vaccine management is for far remote clinics that have no cold chain equipment [26]. Some weaknesses of the study mentioned by the authors include the possibility of an underestimation of cold chain costs and underbudgeted transport costs in the micro plan.

Replacing the current pentavalent vaccine with a thermostable version in a study by Lee et al. [25] demonstrated, without increasing the vaccine price, savings between US$366 and US$10 945 per 100 members of the vaccine's target population. Such cost savings remained even when the vaccine price was doubled or tripled.

### Other benefits

Health workers have also echoed their preference for using vaccines CTC. In one of the studies, about 98.7% of supervisors and 100% of vaccinators indicated that they would prefer to conduct their next vaccination campaign using the CTC strategy [5]. Although the sample selected was not nationally representative, supervisors and vaccinators mentioned some of the top benefits of using the CTC to include more people getting vaccinated per day, lighter vaccine carriers, no need to freeze icepacks, and health workers remaining in the field to continue vaccination without needing to return to base for cold chain storage.

The study by Zipursky et al. [15] concluded that the use of CTC-based strategy will allow for vaccination to happen in areas where there is not reliable cold chain storage capacity, with added benefits to include an allowance for health workers to visit homes in both the early mornings and late afternoons to reach children who were absent during the routine immunization sessions. In this study, health workers were comfortable with the strategy, but some parents showed concern. Lee and Cakouros showed a net positive effect on the supply chain if vaccines are made thermostable, thereby increasing vaccine availability, reduce cold chain requirements and supply chain bottlenecks[16].

### Challenges

Some challenges with CTC vaccines as mentioned by health workers included difficulties with reading the indicator and managing the number of vaccines that should be taken out of the refrigerator at a time [17]. Parental concern was also highlighted.

## DISCUSSIONS

In this scoping review, out of the 173 articles identified through our search 13 met our inclusion criteria and were included in the review. Overall, findings showed the actual and potential benefits of thermostable vaccines, storing vaccines outside the cold chain (OCC), or the use of vaccines approved for controlled temperature chain (CTC) in low- and middle-income countries. Some of the studies showed increased vaccination coverage when vaccines are used outside the cold chain or CTC [16,21-24], while others showed the same seroprotection in vaccines stored OCC or CTC as compared to those stores at standard cold chain temperatures [18]. The increase in vaccination coverage for both CTC and OCC was significant and in some cases 2 to 3 fold higher. Increasing vaccine coverage remains a top priority of the global public health agenda, particularly with emerging and reemerging infectious diseases. Vaccine coverage has stagnated in most countries, with demonstrable equity concerns around the global vaccination coverage [29,30]. Most populations not reached by vaccines are from low- and middle-income countries, remote rural, conflict-affected areas, and peri-urban slums [30]. Even with high immunization coverage, population immunity can only be achieved when the vaccines are still potent at the point of administration. The findings from these studies and numerous other studies demonstrate vaccine potency will not be compromised if conditions are adhered to strictly in CTC or OCC [18,20,22-24].

Some of the studies were focused on the economic benefits of storing vaccines OCC or CTC. These studies showed economic benefits and cost savings when vaccines were kept OCC or CTC [19,25,26], thus signifying these strategies as being cost-effective and could free up resources to reach more children and susceptible persons with lifesaving vaccines or other public health interventions. Stakeholders across Africa and the Western Pacific are ready to adopt HepB vaccine labeled CTC at the right vaccine pricing [31]. As such, vaccine costs and pricing will be key determinants of the success or otherwise of the OCC or CTC adoption.

While vaccines are stored outside the cold chain or in CTC, it is important to safeguard the vaccines from persistent exposure to high ambient temperatures. Temperature swings above 40°C were rare and few vaccines were discarded due to VVM change from extremes of temperature [15,18,22,23]. Vaccines approved CTC has temperature exposure limits as one of the conditions to use the vaccine CTC for a few days. This is meant to protect the vaccines and safeguard potency. Even though some vaccines are stable at ambient temperatures for up to 4 weeks, the use of vaccines CTC usually allows that to happen for a few days in ambient temperatures less than 40°C.

Most of the studies did not report a high vaccine wastage rate when vaccines were stored OCC or CTC, except one which indicated high vaccine wastage attributed to the small sparse population with no baseline to compare whether the pre-intervention period had a similarly high vaccine wastage rate or not [23]. Ensuring vaccines are not getting wasted by a new strategy is crucial in conserving scarce resources. Other benefits noted from using vaccines OCC or CTC included convenience and preference by almost all health workers surveyed in the studies, the potential for more people getting vaccinated per day, lighter vaccine carriage weight, no need to freeze ice-packs, and health workers can stay out in the field and continue vaccination without needing to return to base for cold chain storage [21]. It’s also surmised that the use of CTC-based strategy will allow for vaccination to happen in areas where there is no reliable cold chain storage capacity, with added benefits to include an allowance for health workers to visit homes in both the early mornings and late afternoons to reach children who were absent during the routine immunization sessions [15]. These are factors that play an important role in getting more children vaccinated and reaching the last mile in immunization coverage. These benefits are most significant in low resource settings have limited investments in the immunization supply chain but could also be beneficial in more advanced settings. These benefits were also highlighted by expert opinions who also mentioned that CTC cannot be successful without global guidelines and policies which should be adopted by countries [32].

Shrivastava and Gupta however, in their study showed VVM to be an unreliable indicator of vaccine “potency” at high temperatures[17]. But the drawback of their study was that no laboratory confirmation of vaccine potency was conducted. Some of the challenges reported included apprehension by parents and difficulties with reading the indicator and managing the number of vaccines that should be taken out of the refrigerator at one time.

There are currently many potentially thermostable vaccines or exploration of the feasibility for using these vaccines CTC in the pipeline including Cholera vaccines [33-38], rotavirus vaccines [39,40], inactivated polio vaccines [41], influenza vaccines [42], measles vaccines [43], Human Papilloma Virus (HPV) vaccines including Gardasil spray [44,45], and vaccines as a microneedle patch or spray [42,44,46-48]. Once these vaccines are approved for use CTC, the landscape of immunization will change for the better. To facilitate the effective and proper use of vaccines licensed CTC, operational guidance documents should be expanded and disseminated to policymakers, key stakeholders, and health workers. While frequently asked questions and MenAfriVac specific materials have been developed by WHO [49], they need to be expanded into a full comprehensive operational document and training materials for health workers. The anxiety expressed by some health workers and parents could be managed with frontline health workers receiving both operational and Interpersonal communication (IPC) training on the use of vaccines outside the cold chain or in a controlled temperature chain.

A number of the studies in this review used non-representative samples, with one study by Breakwell et al. having a small sample size [20-24]. However, the studies showed robust methodologies with high internal validity. It is mentioned that even narrowly defined study samples could produce generalizable data if the study is conducted with robust methodology and high internal validity such as the case seen in the 1954 article by Doll and Hill associating cigarette smoking with lung cancer among British physicians [50]. Some of the studies used assumptions that could have oversimplified reality [25,26]. While this is true, there are limits to replicating reality in experiments. But such models do provide good evidence and are recommended to be included in policy and guidelines developed by the WHO [51]. Besides, Mvundura and colleagues [26] concluded that the strongest case for the use of CTC in vaccine management should be for the far remote clinics with no cold chain equipment. It should be noted that many of the clinic settings and particularly the remote and hard-to-reach areas where children have not been vaccinated do also have cold chain issues, as such the CTC may suit their needs. However, maintaining a vaccine in two different storage systems within a country may be confusing and thus may create more harm than good, therefore applying a uniform policy to store a vaccine across a country should be preferable. In one of the studies conducted using oral polio vaccines in OCC by Zipursky et al. [15], a vaccine from a single batch and manufacturer was used for the study. While this study adds to the body of evidence showcasing the benefit of using vaccines OCC or CTC, further studies will need to be conducted specifically for the use of oral polio vaccines OCC or CTC. Currently, there is no policy recommendation on the use of oral polio vaccines OCC by the WHO even as Zipursky et al. [15] studied OCC using oral polio vaccines. More studies might be beneficial in this regard.

The decade of vaccine collaboration through the Global Vaccine Action Plan had an ambitious target of getting (more) vaccines licensed for use at controlled temperature chains – storage outside the recommended +2 to +8°C storage by 2020 [9]. Three vaccines namely; MenAfriVac (40°C for 4 days), PCV13 (40°C for 3 days), and the HPV vaccine Gardasil (42°C for 3 days) were approved CTC [53]; but PCV13 approval for CTC was removed in 2016 to allow consistent labeling across PCV13 products now leaving only two vaccines – MenAfriVac and Gardasil fully CTC prequalified and approved by the World Health Organization [49]. MenAfriVac is being used extensively in the meningitis belt with success - often achieving very high vaccination coverage and preventing meningitis type A outbreaks [20,26,54,55]. The use of HPV Gardasil vaccines CTC is yet to get traction, and more needs to be done to create awareness and facilitate countries to use these vaccines CTC under recommended conditions [49]. HepB vaccine and TT were used OCC and shows great promise. Several vaccines such as Cholera with prospective CTC applications are in the pipeline, and hopefully should be ready for use in the next decade to save lives. Further advancing this agenda, criteria for prequalifying vaccines as extended controlled temperature chain (ECTC) where vaccines can be stored at higher temperatures and more number of days than in CTC are being developed [49]. Although the four priority vaccines considered in the CTC roadmap may not have been fully licensed as CTC [9,49], there is significant progress towards making these and many more vaccines CTC. While no strategy is perfect, the benefits of storing and using vaccines OCC or CTC by far outweigh the risks.

### Limitations

The search was limited to three databases due to their broad coverage of global health sciences including vaccination. Few articles may have been missed in this review; however, we remain confident that is not the case. All original articles with relevance to the topic were included in the review. The scope of this article is the review of studies from low- and middle-income countries to impact global policymaking and may not apply to other settings.

### Public health implications

During the decade of vaccines, significant progress was made with three vaccines so far licensed for CTC use (although one was removed), with only one of the four mentioned in the CTC strategy licensed. With several vaccines being tested OCC and those in various stages of clinical development primarily as thermostable vaccines, the ambitious targets of the global public health community, getting more vaccines used CTC is feasible very shortly. This will change the immunization landscape as it is known today, particularly in low- and middle-income countries. With proper operational guidance dissemination and training for health workers on the proper use of CTC vaccines, more people, particularly children will be reached with cost-effective vaccines in low- and middle-income countries where there remain significant logistical barriers in vaccine storage and delivery. Currently, countries still invest huge amounts of resources both human and material in establishing, sustaining, or even expanding their cold chain capacity to meet the needs of vaccine storage in the cold chain. All these investments have still not sufficed. More vaccines are being introduced necessitating further cold chain capacity expansion, further straining the available resources, especially in LMICs. We continue to advocate and hope that more will be done to accelerate the progress with getting more vaccines licensed and used CTC.

### Recommendations for future research

The use of the vaccine OCC or CTC is beneficial. Accelerating research to get more vaccine storable at room temperature will be very impactful for immunization across LMICs. Also, studies on getting vaccines with multiple antigens thermostable, such as Penta- and Hexa- vaccines, will be beneficial to the world.

## CONCLUSION

So far, two vaccines are approved for use under CTC conditions – MenAfriVac and Gardasil, which are steps in the right direction towards reaching more persons with lifesaving vaccines, particularly in low- and middle-income countries. Storing vaccines outside the cold chain (OCC) or in the controlled temperature chain (CTC) can also be of great value in effectively reaching large target populations over a short period as in epidemic and pandemic situations such as in the case of COVID-19. However, more needs to be done to accelerate research and development and operationalize vaccines already approved CTC to reap the full benefits. This paper provides valuable insight into areas of focus to get more vaccines to be approved and fully utilized under CTC conditions through this comprehensive evidence synthesis.

## Additional material

Online Supplementary Document

## References

[1] Centers for Disease Control and Prevention. Storage best practices for refrigerated vaccines. Atlanta: CDC; 2019.

[2] Weir E Hatch K Preventing cold chain failure: vaccine storage and handling. CMAJ. 2004;171:1050. 10.1503/cmaj.1041565 15505266 PMC526329

[3] Galazka A, Milstien J, Zaffran M. Thermostability of Vaccines. Geneva: WHO Global programme for Vaccines and Immunization; 1998.

[4] World Health Organization. Controlled temperature chain (CTC) - Beyond the traditional cold chain. Geneva: WHO; 2018.

[5] Zipursky S Djingarey MH Lodjo JC Olodo L Tiendrebeogo S Ronveaux O Benefits of using vaccines out of the cold chain: Delivering Meningitis A vaccine in a controlled temperature chain during the mass immunization campaign in Benin. Vaccine. 2014;32:1431-5. 10.1016/j.vaccine.2014.01.038 24559895 PMC5355207

[6] Hipgrave DB Trung NT Vu MH Do TD Nguyen TN Hoang TL Immunogenicity of a locally produced hepatitis B vaccine with the birth dose stored outside the cold chain in rural Vietnam. Am J Trop Med Hyg. 2006;74:255-60. 10.4269/ajtmh.2006.74.255 16474080

[7] Purssell E Reviewing the importance of the cold chain in the distribution of vaccines. Br J Community Nurs. 2015;20:481-6. 10.12968/bjcn.2015.20.10.481 26418400

[8] World Health Organization. Decade of Vaccines - Global Vaccine Action Plan 2011 - 2020. Geneva: WHO; 2012.

[9] World Health Organization. Global Vaccines Action Plan 2011 - 2020. Geneva: WHO; 2013.

[10] Kristensen DD Lorenson T Bartholomew K Villadiego S Can thermostable vaccines help address cold-chain challenges? Results from stakeholder interviews in six low- and middle-income countries. Vaccine. 2016;34:899-904. 10.1016/j.vaccine.2016.01.001 26778422 PMC4744085

[11] Peters MDJ Marnie C Tricco AC Pollock D Munn Z Alexander L Updated methodological guidance for the conduct of scoping reviews. JBI Evidence Synthesis. 2020;18:2119-26. 10.11124/JBIES-20-00167 33038124

[12] Arksey H O’Malley L Scoping studies: towards a methodological framework. Int J Soc Res Methodol. 2005;8:19-32. 10.1080/1364557032000119616

[13] Tricco AC Lillie E Zarin W O’Brien KK Colquhoun H Levac D PRISMA Extension for Scoping Reviews (PRISMA-ScR): Checklist and Explanation. Ann Intern Med. 2018;169:467-73. 10.7326/M18-0850 30178033

[14] Tricco AC Lillie E Zarin W O’Brian KK Colquhoun H Levac D PRISMA-S: An Extension to the PRISMA Statement for Reporting Literature Searches in Systematic Reviews. Ann Int Med. 2019;169:467-73.

[15] Zipursky S Boualam L Cheikh DO Fournier-Caruana J Hamid D Janssen M Assessing the potency of oral polio vaccine kept outside of the cold chain during a national immunization campaign in Chad. Vaccine. 2011;29:5652-6. 10.1016/j.vaccine.2011.06.011 21699946

[16] Lee BY Cakouros BE Assi TM Connor DL Welling J Kone S The impact of making vaccines thermostable in Niger’s vaccine supply chain. Vaccine. 2012;30:5637-43. 10.1016/j.vaccine.2012.06.087 22789507 PMC3592976

[17] Shrivastava A Gupta N Upadhyay P Puliyel J Caution needed in using oral polio vaccine beyond the cold chain: vaccine vial monitors may be unreliable at high temperatures. Indian J Med Res. 2012;135:520-2. 22664500 PMC3385236

[18] Juan-Giner A Domicent C Langendorf C Roper MH Baoundoh P Fermon F A cluster randomized non-inferiority field trial on the immunogenicity and safety of tetanus toxoid vaccine kept in controlled temperature chain compared to cold chain. Vaccine. 2014;32:6220-6. 10.1016/j.vaccine.2014.09.027 25261378

[19] Lydon P Zipursky S Tevi-Benissan C Djingarey MH Gbedonou P Youssouf BO Economic benefits of keeping vaccines at ambient temperature during mass vaccination: the case of meningitis A vaccine in Chad. Bull World Health Organ. 2014;92:86-92. 10.2471/BLT.13.123471 24623901 PMC3949534

[20] Steffen C Tokplonou E Jaillard P Dia R Alladji MNB Gessner B A field based evaluation of adverse events following menafrivac® vaccine delivered in a controlled temperature chain (CTC) approach in benin. Pan Afr Med J. 2014;18:344. 25574320 10.11604/pamj.2014.18.344.3975 PMC4282804

[21] Zipursky S Djingarey MH Lodjo JC Olodo L Tiendrebeogo S Ronveaux O Benefits of using vaccines out of the cold chain: delivering meningitis A vaccine in a controlled temperature chain during the mass immunization campaign in Benin. Vaccine. 2014;32:1431-5. 10.1016/j.vaccine.2014.01.038 24559895 PMC5355207

[22] Kolwaite AR Xeuatvongsa A Ramirez-Gonzalez A Wannemuehler K Vongxay V Vilayvone V Hepatitis B vaccine stored outside the cold chain setting: a pilot study in rural Lao PDR. Vaccine. 2016;34:3324-30. 10.1016/j.vaccine.2016.03.080 27040399 PMC8735871

[23] Breakwell L Anga J Dadari I Sadr-Azodi N Ogaoga D Patel M Evaluation of storing hepatitis B vaccine outside the cold chain in the Solomon Islands: Identifying opportunities and barriers to implementation. Vaccine. 2017;35:2770-4. 10.1016/j.vaccine.2017.04.011 28431814 PMC5893327

[24] Landoh DE Kahn AL Lacle A Adjeoda K Saka B Yaya I Impact of controlled temperature chain (CTC) approach on immunization coverage achieved during the preventive vaccination campaign against meningitis a using menafrivac in Togo in 2014. Pan Afr Med J. 2017;27:38. 28761614 10.11604/pamj.2017.27.38.11873 PMC5516651

[25] Lee BY Wedlock PT Haidari LA Elder K Potet J Manring R Economic impact of thermostable vaccines. Vaccine. 2017;35:3135-42. 10.1016/j.vaccine.2017.03.081 28455169 PMC5547751

[26] Mvundura M Lydon P Gueye A Diaw IK Landoh DE Toi B An economic evaluation of the controlled temperature chain approach for vaccine logistics: evidence from a study conducted during a meningitis A vaccine campaign in Togo. Pan Afr Med J. 2017;27:27. 10.11604/pamj.supp.2017.27.3.12087 29296162 PMC5745944

[27] Coldiron ME Guindo O Makarimi R Soumana I Matar Seck A Garba S Safety of a heat-stable rotavirus vaccine among children in Niger: Data from a phase 3, randomized, double-blind, placebo-controlled trial. Vaccine. 2018;36:3674-80. 10.1016/j.vaccine.2018.05.023 29752026

[28] Munn Z Peters MDJ Stern C Tufanaru C McArthur A Aromataris E Systematic review or scoping review? Guidance for authors when choosing between a systematic or scoping review approach. BMC Med Res Methodol. 2018;18:143. 10.1186/s12874-018-0611-x 30453902 PMC6245623

[29] Peck M Gacic-Dobo M Diallo MS Nedelec Y Sodha SV Wallace AS Global Routine Vaccination Coverage, 2018. MMWR Morb Mortal Wkly Rep. 2019;68:937-42. 10.15585/mmwr.mm6842a1 31647786 PMC6812836

[30] Chopra M Bhutta Z Chang Blanc D Checchi F Gupta A Lemango ET Addressing the persistent inequities in immunization coverage. Bull World Health Organ. 2020;98:146-8. 10.2471/BLT.19.241620 32015586 PMC6986232

[31] Petit D Tevi-Benissan C Woodring J Hennessey K Kahn AL Countries’ interest in a hepatitis B vaccine licensed for the controlled temperature chain; survey results from African and Western Pacific regions. Vaccine. 2017;35:6866-71. 10.1016/j.vaccine.2017.10.025 29132994 PMC5722051

[32] Kahn AL Kristensen D Rao R Extending supply chains and improving immunization coverage and equity through controlled temperature chain use of vaccines. Vaccine. 2017;35:2214-6. 10.1016/j.vaccine.2016.10.091 28364934

[33] Grandesso F Rafael F Chipeta S Alley I Saussier C Nogareda F Oral cholera vaccination in hard-to-reach communities, Lake Chilwa, Malawi. Bull World Health Organ. 2018;96:817-25. 10.2471/BLT.17.206417 30505029 PMC6249704

[34] Islam MT Chowdhury F Qadri F Sur D Ganguly NK Trials of the killed oral cholera vaccine (Shanchol) in India and Bangladesh: Lessons learned and way forward. Vaccine. 2020. Online ahead of print. 10.1016/j.vaccine.2019.06.082 31301917

[35] Massing LA Aboubakar S Blake A Page AL Cohuet S Ngandwe A Highly targeted cholera vaccination campaigns in urban setting are feasible: The experience in Kalemie, Democratic Republic of Congo. PLoS Negl Trop Dis. 2018;12: e0006369. 10.1371/journal.pntd.0006369 29734337 PMC5957443

[36] Qadri F Oral cholera vaccine studies in high cholera endemic settings in bangladesh. Am J Trop Med Hyg. 2016;95:5.26711520

[37] Saha A Khan A Bhuiyan TR Clemens JD Qadri F Shanchol, the oral cholera vaccine is safe and immunogenic when stored at elevated temperatures in bangladeshi participants. Am J Trop Med Hyg. 2016;95:133-4.

[38] Saha A Khan A Salma U Jahan N Bhuiyan TR Chowdhury F The oral cholera vaccine Shanchol when stored at elevated temperatures maintains the safety and immunogenicity profile in Bangladeshi participants. Vaccine. 2016;34:1551-8. 10.1016/j.vaccine.2016.02.020 26896684

[39] Asowata OE Ashiru OT Sturm AW Moodley P Stability of a monovalent rotavirus vaccine after exposure to different temperatures observed in KwaZulu-Natal, South Africa. Afr Health Sci. 2019;19:1993-9. 10.4314/ahs.v19i2.22 31656482 PMC6794501

[40] Madan M Sikriwal D Sharma G Shukla N Mandyal AK Kale S Rational design of heat stable lyophilized rotavirus vaccine formulations. Hum Vaccin Immunother. 2018;14:2132-41. 10.1080/21645515.2018.1487499 29953317 PMC6183320

[41] Kraan H Ploemen I van de Wijdeven G Que I Lowik C Kersten G Alternative delivery of a thermostable inactivated polio vaccine. Vaccine. 2015;33:2030-7. 10.1016/j.vaccine.2015.03.011 25772676

[42] Esser ES Pulit-Penaloza JA Kalluri H McAllister D Vassilieva EV Littauer EQ Microneedle patch delivery of influenza vaccine during pregnancy enhances maternal immune responses promoting survival and long-lasting passive immunity to offspring. Sci Rep. 2017;7:5705. 10.1038/s41598-017-05940-7 28720851 PMC5515933

[43] Hien ND Luan LT Huong NT Thu NNA Thuy TH Hoa NX High immunogenicity of measles AIK-C vaccine produced in Vietnam. East J Med. 2011;16:199-207.

[44] Kunda NK Peabody J Zhai LK Price DN Chackerian B Tumban E Evaluation of the thermal stability and the protective efficacy of spray-dried HPV vaccine, Gardasil (R) 9. Hum Vaccin Immunother. 2019;15:1995-2002. 10.1080/21645515.2019.1593727 30883270 PMC6746511

[45] Seitz H Ribeiro-Muller L Canali E Bolchi A Tommasino M Ottonello S Robust In Vitro and In Vivo Neutralization against Multiple High-Risk HPV Types Induced by a Thermostable Thioredoxin-L2 Vaccine. Cancer Prev Res (Phila). 2015;8:932-41. 10.1158/1940-6207.CAPR-15-0164 26170394

[46] Arya J Prausnitz MR Microneedle patches for vaccination in developing countries. J Control Release. 2016;240:135-41. 10.1016/j.jconrel.2015.11.019 26603347 PMC4871790

[47] Jacoby E Jarrahian C Hull HF Zehrung D Opportunities and challenges in delivering influenza vaccine by microneedle patch. Vaccine. 2015;33:4699-704. 10.1016/j.vaccine.2015.03.062 25842218

[48] Marshall S Sahm LJ Moore AC The success of microneedle-mediated vaccine delivery into skin. Hum Vaccin Immunother. 2016;12:2975-83. 10.1080/21645515.2016.1171440 27050528 PMC5137519

[49] World Health Organization. CONTROLLED TEMPERATURE CHAIN: Strategic Roadmap for Priority Vaccines 2017-2020. 2017. Available: https://apps.who.int/iris/bitstream/handle/10665/272994/WHO-IVB-17.20-eng.pdf. Accessed 10 May 2020.

[50] Kukull WA Ganguli M Generalizability: the trees, the forest, and the low-hanging fruit. Neurology. 2012;78:1886-91. 10.1212/WNL.0b013e318258f812 22665145 PMC3369519

[51] Egger M Johnson L Althaus C Schoni A Salanti G Low N Developing WHO guidelines: Time to formally include evidence from mathematical modelling studies. F1000Res. 2017;6:1584. 10.12688/f1000research.12367.1 29552335 PMC5829466

[52] Zhang J He PF Liang ZL Xu M Expanded strategy for cold chain supply of vaccines: Controlled temperature chain. Zhongguo Shengwuzhipinxue Zazhi. 2018;31:1036-9.

[53] World Health Organization. The controlled temperature chain: frequently asked questions. Geneva: WHO; 2016.

[54] Bwaka A Bita A Lingani C Fernandez K Durupt A Mwenda JM Status of the Rollout of the Meningococcal Serogroup A Conjugate Vaccine in African Meningitis Belt Countries in 2018. J Infect Dis. 2019;220:S140-7. 10.1093/infdis/jiz336 31671448 PMC6822965

[55] Landoh DE Kahn AL Lacle A Adjeoda K Saka B Yaya I Impact of Controlled Temperature Chain (CTC) approach on immunization coverage achieved during the preventive vaccination campaign against meningitis A using MenAfriVac in Togo in 2014. Pan Afr Med J. 2017;27:38. 28761614 10.11604/pamj.2017.27.38.11873 PMC5516651

