# Supplementary material for: How the use of vaccines outside the cold chain or in controlled temperature chain contributes to improving immunization coverage in low- and middle-income countries (LMICs): A scoping review of the literature
Source: J Glob Health. 2021 Jan 31;11:04004. doi: 10.7189/jogh.11.04004 (PMC7915947; doi:10.7189/jogh.11.04004)
Supplement: Online Supplementary Document [file jogh-11-04004-s001.pdf]

Appendix S1

Preferred Reporting Items for Systematic reviews and Meta-Analyses extension for Scoping Reviews (PRISMA-ScR) Checklist

SECTION	ITEM	PRISMA-ScR CHECKLIST ITEM	REPORTED ON PAGE #
**TITLE**			
Title	1	Identify the report as a scoping review.	1
**ABSTRACT**			
Structured summary	2	Provide a structured summary that includes (as applicable): background, objectives, eligibility criteria, sources of evidence, charting methods, results, and conclusions that relate to the review questions and objectives.	3
**INTRODUCTION**			
Rationale	3	Describe the rationale for the review in the context of what is already known. Explain why the review questions/objectives lend themselves to a scoping review approach.	5 & 6
Objectives	4	Provide an explicit statement of the questions and objectives being addressed with reference to their key elements (e.g., population or participants, concepts, and context) or other relevant key elements used to conceptualize the review questions and/or objectives.	7
**METHODS**			
Protocol and registration	5	Indicate whether a review protocol exists; state if and where it can be accessed (e.g., a Web address); and if available, provide registration information, including the registration number.	7
Eligibility criteria	6	Specify characteristics of the sources of evidence used as eligibility criteria (e.g., years considered, language, and publication status), and provide a rationale.	8
Information sources*	7	Describe all information sources in the search (e.g., databases with dates of coverage and contact with authors to identify additional sources), as well as the date the most recent search was executed.	8
Search	8	Present the full electronic search strategy for at least 1 database, including any limits used, such that it could be repeated.	8 and Appendix S3
Selection of sources of evidence†	9	State the process for selecting sources of evidence (i.e., screening and eligibility) included in the scoping review.	9 & 10

SECTION	ITEM	PRISMA-ScR CHECKLIST ITEM	REPORTED ON PAGE #
Data charting process‡	10	Describe the methods of charting data from the included sources of evidence (e.g., calibrated forms or forms that have been tested by the team before their use, and whether data charting was done independently or in duplicate) and any processes for obtaining and confirming data from investigators.	10
Data items	11	List and define all variables for which data were sought and any assumptions and simplifications made.	10
Critical appraisal of individual sources of evidence§	12	If done, provide a rationale for conducting a critical appraisal of included sources of evidence; describe the methods used and how this information was used in any data synthesis (if appropriate).	Not done
Synthesis of results	13	Describe the methods of handling and summarizing the data that were charted.	10
**RESULTS**			
Selection of sources of evidence	14	Give numbers of sources of evidence screened, assessed for eligibility, and included in the review, with reasons for exclusions at each stage, ideally using a flow diagram.	33
Characteristics of sources of evidence	15	For each source of evidence, present characteristics for which data were charted and provide the citations.	10 – 11
Critical appraisal within sources of evidence	16	If done, present data on critical appraisal of included sources of evidence (see item 12).	Not done
Results of individual sources of evidence	17	For each included source of evidence, present the relevant data that were charted that relate to the review questions and objectives.	34 – 40
Synthesis of results	18	Summarize and/or present the charting results as they relate to the review questions and objectives.	10 – 18
**DISCUSSION**			
Summary of evidence	19	Summarize the main results (including an overview of concepts, themes, and types of evidence available), link to the review questions and objectives, and consider the relevance to key groups.	19 – 23
Limitations	20	Discuss the limitations of the scoping review process.	24
Conclusions	21	Provide a general interpretation of the results with respect to the review questions and objectives, as well as potential implications and/or next steps.	24 – 25

SECTION	ITEM	PRISMA-ScR CHECKLIST ITEM	REPORTED ON PAGE #
**FUNDING**			
Funding	22	Describe sources of funding for the included sources of evidence, as well as sources of funding for the scoping review. Describe the role of the funders of the scoping review.	26

JBI = Joanna Briggs Institute; PRISMA-ScR = Preferred Reporting Items for Systematic reviews and Meta-Analyses extension for Scoping Reviews.
* Where *sources of evidence* (see second footnote) are compiled from, such as bibliographic databases, social media platforms, and Web sites.
† A more inclusive/heterogeneous term used to account for the different types of evidence or data sources (e.g., quantitative and/or qualitative research, expert opinion, and policy documents) that may be eligible in a scoping review as opposed to only studies. This is not to be confused with *information sources* (see first footnote).
‡ The frameworks by Arksey and O'Malley (6) and Levac and colleagues (7) and the JBI guidance (4, 5) refer to the process of data extraction in a scoping review as data charting.
§ The process of systematically examining research evidence to assess its validity, results, and relevance before using it to inform a decision. This term is used for items 12 and 19 instead of "risk of bias" (which is more applicable to systematic reviews of interventions) to include and acknowledge the various sources of evidence that may be used in a scoping review (e.g., quantitative and/or qualitative research, expert opinion, and policy document).

From: Tricco AC, Lillie E, Zarin W, O'Brien KK, Colquhoun H, Levac D, et al. PRISMA Extension for Scoping Reviews (PRISMAScR): Checklist and Explanation. Ann Intern Med. 2018;169:467–473. doi: 10.7326/M18-0850.

Appendix S2

PRISMA-S Checklist

Section/topic	#	Checklist item	Location(s) Reported
**INFORMATION SOURCES AND METHODS**			
Database name	1	Name each individual database searched, stating the platform for each.	**Page 8**
Multi-database searching	2	If databases were searched simultaneously on a single platform, state the name of the platform, listing all of the databases searched.	**Page 8**
Study registries	3	List any study registries searched.	**Page 8**
Online resources and browsing	4	Describe any online or print source purposefully searched or browsed (e.g., tables of contents, print conference proceedings, web sites), and how this was done.	**Page 8**
Citation searching	5	Indicate whether cited references or citing references were examined, and describe any methods used for locating cited/citing references (e.g., browsing reference lists, using a citation index, setting up email alerts for references citing included studies).	**Page 8**
Contacts	6	Indicate whether additional studies or data were sought by contacting authors, experts, manufacturers, or others.	**Page 9**
Other methods	7	Describe any additional information sources or search methods used.	**Page 9**
**SEARCH STRATEGIES**			
Full search strategies	8	Include the search strategies for each database and information source, copied and pasted exactly as run.	**Appendix S3 & Page 8**
Limits and restrictions	9	Specify that no limits were used, or describe any limits or restrictions applied to a search (e.g., date or time period, language, study design) and provide justification for their use.	**Page 8 & Appendix S3**
Search filters	10	Indicate whether published search filters were used (as originally designed or modified), and if so, cite the filter(s) used.	**Page 8 & Appendix S3**
Prior work	11	Indicate when search strategies from other literature reviews were adapted or reused for a substantive part or all of the search, citing the previous review(s).	**Page 7**

Updates	12	Report the methods used to update the search(es) (e.g., rerunning searches, email alerts).	**Not applicable**
Dates of searches	13	For each search strategy, provide the date when the last search occurred.	**Page 10 & Appendix S3**
**PEER REVIEW**			
Peer review	14	Describe any search peer review process.	**Page 8**
**MANAGING RECORDS**			
Total Records	15	Document the total number of records identified from each database and other information sources.	**Pages 9,10 & Figure 1**
Deduplication	16	Describe the processes and any software used to deduplicate records from multiple database searches and other information sources.	**Page 9**

PRISMA-S: An Extension to the PRISMA Statement for Reporting Literature Searches in Systematic Reviews
Rethlefsen ML, Kirtley S, Waffenschmidt S, Ayala AP, Moher D, Page MJ, Koffel JB, PRISMA-S Group.
Last updated February 27, 2020.

Appendix S3

Search Strategy

**Database 1 PubMed (NCBI)**	((((("outside the cold chain" OR "controlled temperature chain" OR "out of the cold chain" OR "out of cold chain" OR "ambient temperature-stable" OR "ambient temperature" OR "environmental temperature" OR "room temperature storage" OR "room-temperature" OR "temperature-stable" OR "thermostable" OR thermostability OR "hot temperature")) OR hot temperature[MeSH Terms]))) AND ((((("immunization coverage" OR "immunisation coverage" OR "vaccination coverage" OR Vaccin*)) OR vaccination OR immunization[MeSH Terms] OR vaccination[MeSH Terms]) OR universal coverage[MeSH Terms]))) AND ((("low and middle income countries" OR "LMIC" OR "low-income countries" OR "middle-income countries" "developing countries" OR "poor countries" OR "poverty" OR "under-developed countries" OR "low resource" OR "emerging economies" OR "African countries" OR Asia OR Pacific OR "Latin America" OR "Middle East" OR low income population[MeSH Terms] OR low income populations[MeSH Terms] OR developing countries[MeSH Terms] OR developing nations[MeSH Terms] OR less developed countries[MeSH Terms] OR third world countries[MeSH Terms] OR under developed countries[MeSH Terms] OR poverty[MeSH Terms] OR africa[MeSH Terms] OR sub saharan africa[MeSH Terms] OR asia[MeSH Terms] OR latin america[MeSH Terms] OR pacific islands[MeSH Terms] OR arab[MeSH Terms] OR arab countries[MeSH Terms])))) Sort by: Best Match Filters: Publication date from 2011/01/01 to 2020/12/31; Humans
**Dates & Other Limiters Applied** (e.g., Dates (justify), language)	Sort by: Publication date from 2011/01/01 to 2020/12/31<br>Species: Humans<br>The period 2011 – 2020 was declared as the Decade of Vaccines (DoV) and timeline to achive the targets of the Global Vaccine Action Plan (GVAP) 2011 – 2020. This literature review will contribute mainly to strategic objectives 6 of the GVAP by assessing evidence available in support of indicator 6.4. – Number of vaccines that have either been re-licensed or licensed for use in a controlled-temperature chain at temperatures above the traditional 2-8 °C range (WHO, 2013). But also, it will contribute to strategic indicators 5 and 3 of the GVAP to a lesser extent.
**Articles Retrieved (qty)**	70

**Database 2**<br>**EMBASE (Elsevier)**	('outside the cold chain' OR 'controlled temperature chain' OR 'out of the cold chain' OR 'out of cold chain' OR 'ambient temperature-stable' OR 'ambient temperature'/exp OR 'room temperature storage' OR 'room-temperature'/exp OR 'temperature-stable' OR 'thermostable' OR 'hot temperature'/exp OR 'high temperature'/exp OR 'thermostability'/exp OR 'room temperature'/exp OR 'environmental temperature'/exp) AND ('immunization coverage'/exp OR 'immunisation coverage'/exp OR vaccin* OR 'universal coverage'/exp OR (universal AND coverage) OR 'immunization'/exp OR 'vaccination coverage'/exp OR 'vaccination'/exp OR 'vaccine'/exp) AND ('low and middle income countries' OR 'lmic' OR 'low-income countries' OR 'middle-income countries' OR 'developing countries'/exp OR 'developing nations' OR 'poor countries' OR 'under-developed countries' OR 'low resource' OR 'emerging economies' OR 'african countries' OR 'sub saharan africa'/exp OR pacific OR 'latin america'/exp OR 'middle east'/exp OR 'low income population'/exp OR 'low middle income country'/exp OR 'low income country'/exp OR 'middle income country'/exp OR 'developing country'/exp OR 'lowest income group'/exp OR 'poverty'/exp OR

	'africa'/exp OR 'africa south of the sahara'/exp OR 'asia'/exp OR (south AND 'central america'/exp) OR 'pacific islands'/exp) AND [humans]/lim AND [2011-2020]/py
**Dates & Other Limiters Applied**<br>(e.g., Dates (justify), language)	Publication year: 2011 – 2020 (same reason as above)<br>Species: Human<br>Drug delimiter for all vaccines listed was applied: AND ('bcg vaccine'/dd OR 'cholera vaccine'/dd OR 'dengue vaccine'/dd OR 'diphtheria pertussis tetanus vaccine'/dd OR 'hepatitis b vaccine'/dd OR 'human immunodeficiency virus vaccine'/dd OR 'inactivated vaccine'/dd OR 'influenza vaccine'/dd OR 'japanese encephalitis vaccine'/dd OR 'live vaccine'/dd OR 'malaria vaccine'/dd OR 'measles vaccine'/dd OR 'meningococcus vaccine'/dd OR 'oral poliomyelitis vaccine'/dd OR 'pneumococcus vaccine'/dd OR 'poliomyelitis vaccine'/dd OR 'rabies vaccine'/dd OR 'rotavirus vaccine'/dd OR 'tetanus toxoid'/dd OR 'unclassified drug'/dd OR 'vaccine'/dd OR 'virus vaccine'/dd OR 'yellow fever vaccine'/dd OR 'aik c vaccine' OR 'typhoid vaccine'/dd OR 'unindexed drug'/dd)
**Articles Retrieved (qty)**	67

**Database 3**<br>**Web of Science (Clarivate Analytics)**	ALL=((("outside the cold chain" OR "controlled temperature chain" OR "out of the cold chain" OR "out of cold chain" OR "ambient temperature-stable" OR "ambient temperature" OR "environmental temperature" OR "room temperature storage" OR "room-temperature" OR "temperature-stable" OR "thermostable" OR "thermostability" OR "hot temperature")) AND (("immunization coverage" OR "immunisation coverage" OR "vaccination coverage" OR Vaccin* OR vaccination OR immunization OR "universal coverage")) AND (("low and middle income countries" OR "LMIC" OR "low-income countries" OR "middle-income countries" "developing countries" OR "developing nations" OR "poor countries" OR "poverty" OR "under-developed countries" OR "low resource" OR "emerging economies" OR "African countries" "sub saharan Africa" OR Asia OR Pacific OR "Latin America" OR "Middle East" OR "low income population")))
**Dates & Other Limiters Applied**<br>(e.g., Dates (justify), language)	Refined by: PUBLICATION YEARS: (2019 OR 2014 OR 2018 OR 2013 OR 2017 OR 2012 OR 2016 OR 2011 OR 2015)<br>(Justification same as above)
**Articles Retrieved (qty)**	36
